# The integration of differentially expressed genes based on multiple microarray datasets for prediction of the prognosis in oral squamous cell carcinoma

**DOI:** 10.1080/21655979.2021.1947076

**Published:** 2021-07-05

**Authors:** Yinuan Zhao, Jiacheng Huang, Jianzhi Chen

**Affiliations:** a School of Stomatology, Zhejiang Chinese Medical University, Hangzhou, Zhejiang, China; b School of Medicine, Zhejiang University, Hangzhou, Zhejiang, China

**Keywords:** Oral squamous cell carcinoma, biomarker, GEO, TCGA, bioinformatics, prognosis

## Abstract

Oral squamous cell carcinoma (OSCC) is a common human malignancy. However, its pathogenesis and prognostic information are poorly elucidated. In the present study, we aimed to probe the most significant differentially expressed genes (DEGs) and their prognostic performance in OSCC. Multiple microarray datasets from the Gene Expression Omnibus (GEO) database were aggregated to identify DEGs between OSCC tissue and control tissue. Least absolute shrinkage and selection operator (LASSO) Cox model was constructed to determine the prognostic performance of the aggregated DEGs based on The Cancer Genome Atlas (TCGA) OSCC cohort. Ten datasets with 341 OSCC samples and 283 control samples were included. Kyoto Encyclopedia of Genes and Genomes (KEGG) pathway enrichment revealed that the integrated DEGs were enriched in the IL-17 signaling pathway, viral protein interactions with cytokines and cytokine receptors, and amoebiasis, among others. Our LASSO Cox model was able to discriminate two groups with different overall survival in the training cohort and test cohort (p < 0.001). The time-dependent receiver operating characteristic (ROC) curve revealed that the area under the curve (AUC) values at one year, three years, and five years were 0.831, 0.898, and 0.887, respectively. In the testing cohort, the time-dependent ROC curve showed that the AUC values at one year, three years, and five years were 0.696, 0.693, and 0.860, respectively. Our study showed that the integrated DEGs of OSCC might be applicable in the evaluation of prognosis in OSCC. However, further research should be performed to validate our findings.

## Graphical abstract

**Figure:**
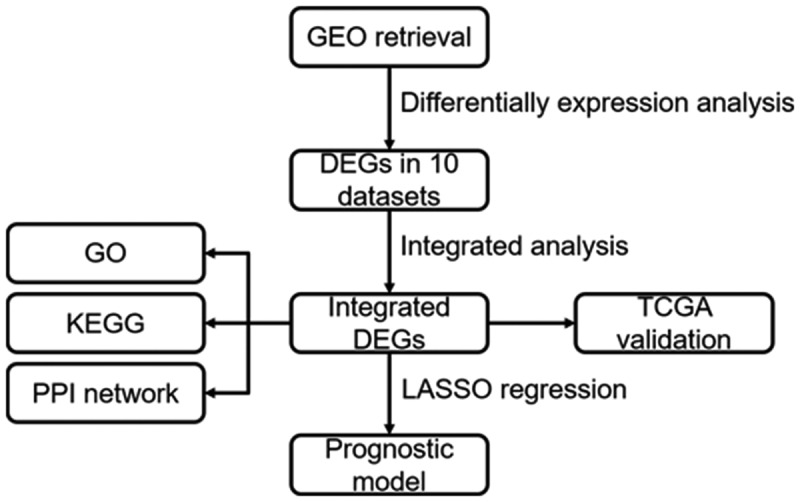

## Introduction

Oral squamous cell carcinoma (OSCC), which constitutes 90% of all oral cancer-related deaths, is a common human malignancy worldwide. More than 370,000 new cases are diagnosed each year, and it is particularly prevalent in Melanesia and South Central Asia [1]. OSCC occurs anywhere in the oral cavity, including the tongue, gingiva, oral floor, palate, and buccal mucosa [2]. Its etiology involves many risk factors, including tobacco, alcohol, and viruses. Despite some new advances in diagnosis and multimodal therapies, the 5-year survival rate of OSCC patients only reaches 50% due to distant metastases, locoregional relapse and second primary tumor. Many remarkable advances have been achieved for OSCC treatment in recent years, such as mainstream surgery, radiotherapy, chemotherapy, immunotherapy, and gene therapy, which are currently undergoing research. For instance, PD-1 blockade has been applied for OSCC treatment, and pertinent studies are being performed to further understand the side effects and medication indications. In addition, preliminary clinical trials of gene therapy, as well as in combination with traditional therapy, showed that gene therapy might be a promising alternative strategy for OSCC treatment [3].

The pathogenesis of OSCC is likely multifactorial and has not been fully elucidated. Moreover, the prognosis of patients diagnosed with OSCC is poor. The study of the pathogenesis of OSCC contributes to personalized therapies for patients and improves prognosis. We hypothesized that during the pathogenesis of OSCC, dysregulation of genes occurred, which altered the behavior of oral mucosal epithelial cells. Subsequently, the gene regulatory network became disordered, and these dysregulated genes might be potential prognostic indictors of OSCC. To probe the dysregulated genes based on multiple studies and their prognostic performance, in the present study, multiple microarray datasets from the Gene Expression Omnibus (GEO) database were aggregated to acquire differentially expressed genes (DEGs) between OSCC tissue and control tissue. The expression differences of the combined DEGs were validated via The Cancer Genome Atlas (TCGA) RNA-sequence data. Furthermore, Gene Ontology (GO) and Kyoto Encyclopedia of Genes and Genomes (KEGG) analyses were performed based on the integrated DEGs. Finally, least absolute shrinkage and selection operator (LASSO) Cox model was constructed to determine the prognostic performance based on the aggregated DEGs.

## Materials and methods

### Retrieval of GEO microarray datasets

‘Oral squamous cell carcinoma’ was used as a key word for retrieval in the GEO (Gene Expression Omnibus) database (https://www.ncbi.nlm.nih.gov/geo/) (access date: 2020/10/4). Additional restrictions on the retrieval results were as follows: organism (*Homo sapiens*) and study type (expression profiling by array). The inclusion criteria were as follows: 1) studies containing OSCC tissue and control tissue; 2) at least five cases of OSCC and controls; and 3) head and neck squamous cell carcinoma (HNSCC) samples whose sampling location was the oral cavity. The exclusion criteria were as follows: 1) studies containing only OSCC tissue or control tissue; 2) fewer than five cases of OSCC and controls; and 3) studies applying specially designed platforms for a cluster of genes, such as immune profiles.

### Identification of differentially expressed genes in GEO datasets

The expression matrices of all included datasets and their corresponding annotation platforms were downloaded via the ‘GEOquery’ package [4]. All probe names in each dataset were converted into gene symbols according to their corresponding platforms. Genes whose expression value was zero in more than 10% of all samples were eliminated and were not subsequently analyzed. DEGs, their corresponding log2-fold changes (logFC), and p values were acquired using the ‘limma’ package [5]. LogFC here represents the relative gene expression value in tumor samples divided by the relative gene expression value in paracancerous tissue followed by log2 transformation. Volcano plots were drawn based on each dataset using the ‘EnhancedVolcano’ package.

### Integration of differentially expressed genes

All lists of DEGs from the included GEO datasets were ordered according to logFC value. The integrated DEG list was acquired using the ‘RobustRankAggreg’ package [6] and was preserved for the following analysis.

### Validation of DEGs in the TCGA cohort

OSCC samples were selected from the TCGA HNSCC cohort (access data: 2020/11/15). The HTseq count value was downloaded and transformed on a log2 scale for further analysis. All ensemble names in the expression matrix were converted into symbols according to the ‘Homo_sapiens.GRCh38.101.gtf’ file downloaded from the Ensembl database (https://asia.ensembl.org/index.html). Afterward, the expression patterns of the top five upregulated and downregulated genes in the integrated DEG list were explored in TCGA OSCC samples. Receiver operating characteristic (ROC) curves were drawn. An area under the curve (AUC) over 0.9 represents a high accuracy for discrimination. An AUC between 0.7 and 0.9 shows moderate discriminative accuracy. An AUC between 0.5 and 0.7 is regarded as low discriminative capability. An AUC less than 0.5 means that the parameter or target cannot be applied for discrimination at all.

### GO, KEGG enrichment analysis and PPI visualization of the integrated DEGs

GO annotation and KEGG enrichment analysis were performed by using the ‘clusterProfiler’ package [7]. Protein-to-protein interactions (PPIs) were visualized via the Search Tool for the Retrieval of Interacting Genes (STRING version 11.0: https://string-db.org/).

### Construction of prognostic model based on the integrated DEGs

TCGA OSCC samples were randomly divided using the ‘sample’ function in R into a training cohort (2/3) and a test cohort (1/3). Univariate analysis based on the integrated DEGs was performed to screen the prognosis-associated genes. A LASSO Cox regression model was constructed based on the training cohort, and the model was tested in the testing cohort. LASSO regression introduced a penalty function and an adjustable parameter (λ) so that the coefficients of some less important parameters were reduced to zero in the regression model and the model was simplified.

### Statistical analysis

All statistical analyses mentioned in this manuscript were completed in R 4.0.2. Genes with LogFC > 1 and adjusted p value < 0.05 were considered DEGs. The expression of OSCC and the control based on the top five upregulated and downregulated genes in the integrated DEG list was compared by independent-sample t-test. The continuous variables of integrated DEG expression were converted into dichotomous variables when applying the ‘surv_cutpoint’ function in the ‘survminer’ package. The Kaplan-Meier method, also named the product-limit method, was used to elucidate the association between genes and prognosis, and a log rank p value less than 0.05 was considered statistically significant. LASSO Cox regression was performed via the ‘glmnet’ package [8]. Time-dependent ROC curves were drawn to display the prediction power at different time points.

## Results

We assumed that the dysregulated genes were responsible for the pathogenesis of OSCC. First, the GEO database was comprehensively searched. Second, integrated DEGs were identified to explore the dysregulated genes in OSCC, and the representative DEGs were validated via TCGA data. Subsequently, GO and KEGG analyses revealed the biological processes and pathways related to the integrated DEGs, which contributed to understanding the potential mechanism of OSCC. Finally, to evaluate the prognostic performance of these DEGs in OSCC, LASSO regression was applied.

### Screening of the included OSCC GEO datasets

**Figure 1.**
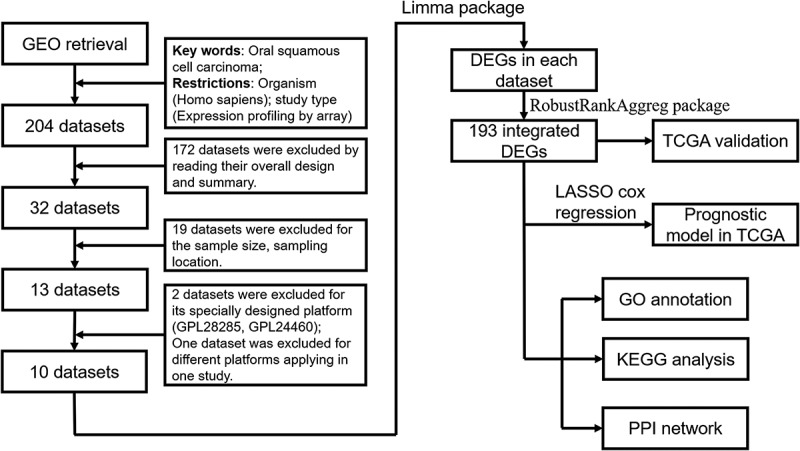
Flowchart of this study design

Figure 1 is a flowchart that displays our study design. A total of 204 GEO datasets were screened. A total of 172 datasets were excluded by reading their overall design and summary. Nineteen datasets were excluded for sample size and sampling location. Two datasets were excluded for a specially designed platform (GPL28285, GPL24460). One dataset was excluded for different platforms applied in one study. Finally, ten datasets with 341 OSCC samples and 283 control samples met the inclusion criteria in this study, and the basic characteristics are listed in Table 1.

**Table 1.** Characteristics of the included datasets of oral squamous cells carcinoma

GEO accession	Year	Country or region	Cases number	Control number	Platform
GSE138206	2019	China	6	12	GPL570
GSE107591	2017	Italy	17	16	GPL6244
GSE74530	2017	USA	6	6	GPL570
GSE75538	2016	India	14	14	GPL18281
GSE37991	2013	Taiwan	40	40	GPL6883
GSE35261	2012	Japan	11	22	GPL8950
GSE31056	2011	USA	23	73	GPL10526
GSE30784	2011	USA	167	62	GPL570
GSE13601	2008	USA	31	26	GPL8300
GSE9844	2008	USA	26	12	GPL570

### Identification of integrated DEGs of OSCC

**Figure 2.**
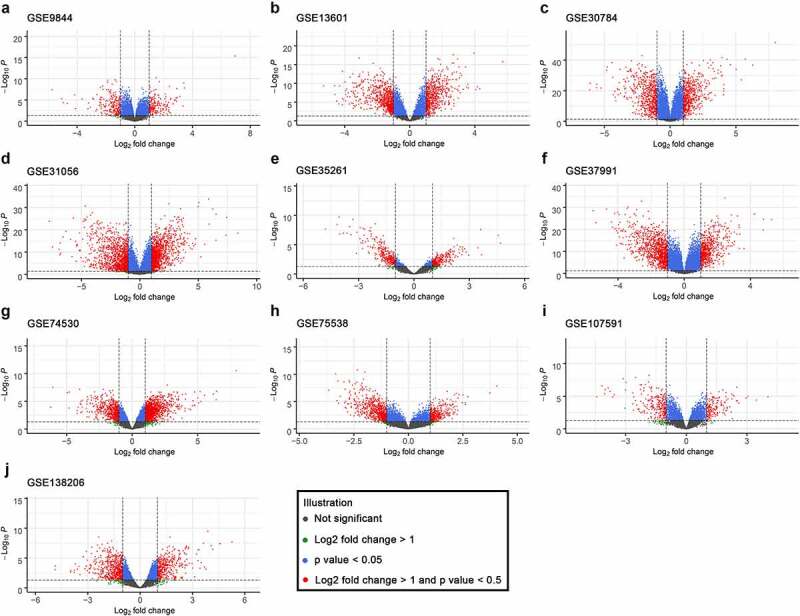
Volcano plots of the ten included GEO datasets revealed differentially expressed genes

**Figure 3.**
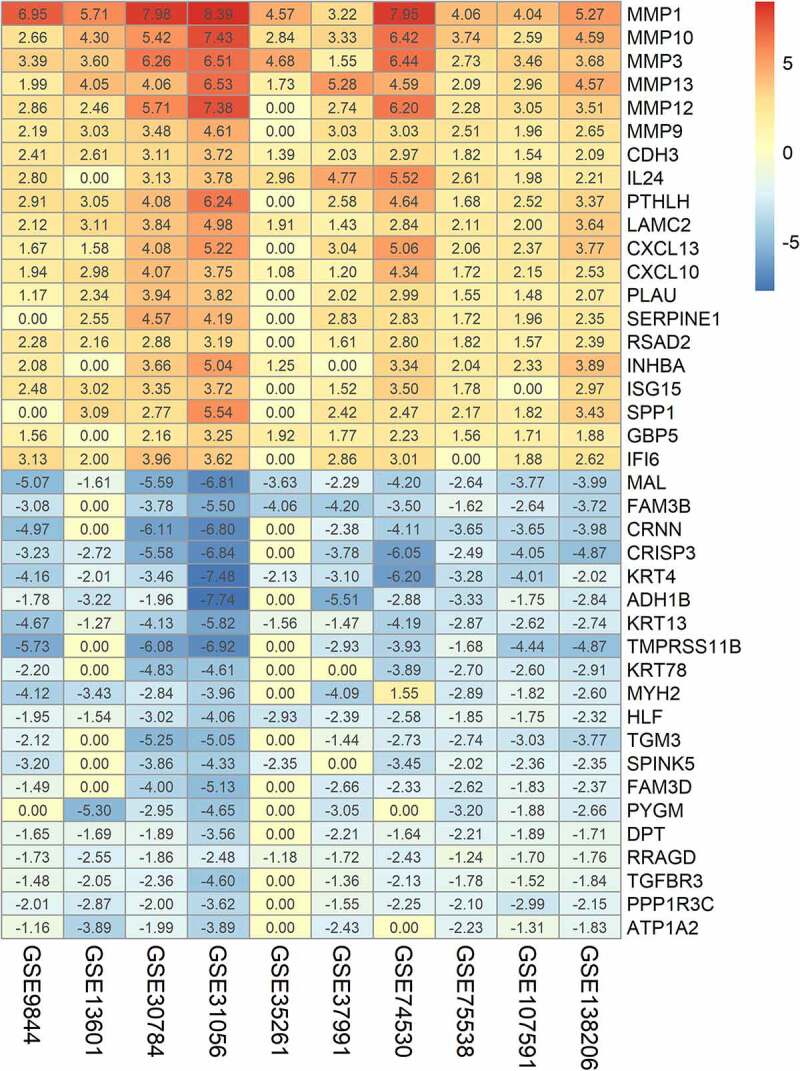
Heatmap of the integrated DEGs based on multiple microarray datasets

The DEGs in ten GEO datasets were acquired via the ‘limma’ package, and the volcano plots are listed in Figure 2. Through the ‘RobustRankAggreg’ package, 193 integrated DEGs were identified, including 93 overexpressed genes and 100 downregulated genes (Supplementary Table 1). The top five upregulated genes were matrix metalloproteinases 1 (MMP1), matrix metalloproteinases 10 (MMP10), matrix metalloproteinases 3 (MMP3), matrix metalloproteinases 13 (MMP13), and matrix metalloproteinases 12 (MMP12), and the top five downregulated genes were cysteine rich secretory protein 3 (CRISP3), T cell differentiation protein (MAL), keratin 4 (KRT4), transmembrane serine protease 11B (TMPRSS11B), and cornulin (CRNN). Additionally, the twenty most significantly upregulated and downregulated genes and their logFC values are displayed in the heatmap (Figure 3).

### Validation of DEGs in TCGA cohort

**Figure 4.**
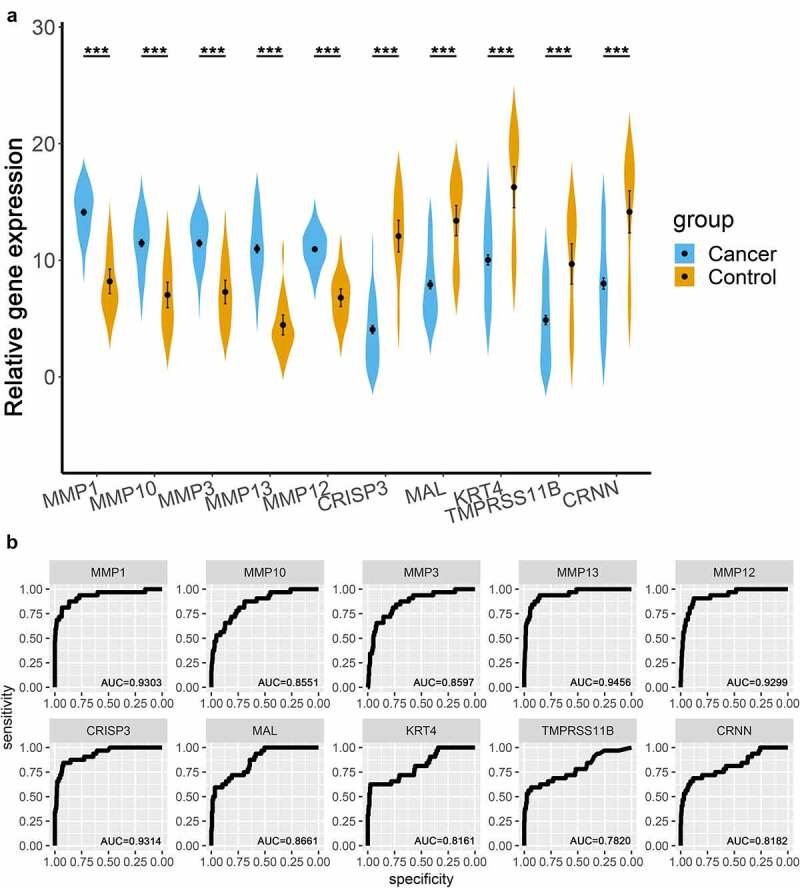
Validation of the top 10 integrated DEGs in the TCGA OSCC cohort. (a). Violin plots, relative gene expression = log2(count+1); (b). ROC curves of the expression of MMP1, MMP10, MMP3, MMP13, MMP12, CRISP3, MAL, KRT4, TMPRSS11B, and CRNN. *** represented that p value < 0.001

We compared the expression of the top five upregulated and downregulated genes between OSCC and controls in TCGA. All expression levels of the ten genes were significantly different between OSCC and the control (Figure 4(a), p < 0.001). The AUC values of MMP1, MMP10, MMP3, MMP13, MMP12, CRISP3, MAL, KRT4, TMPRSS11B, and CRNN in the ROC curves were 0.9303, 0.8551, 0.8579, 0.9456, 0.9299, 0.9314, 0.8661, 0.8161, 0.7820, and 0.8182, respectively (Figure 4(b)).

### GO and KEGG analysis of the integrated DEGs

**Figure 5.**
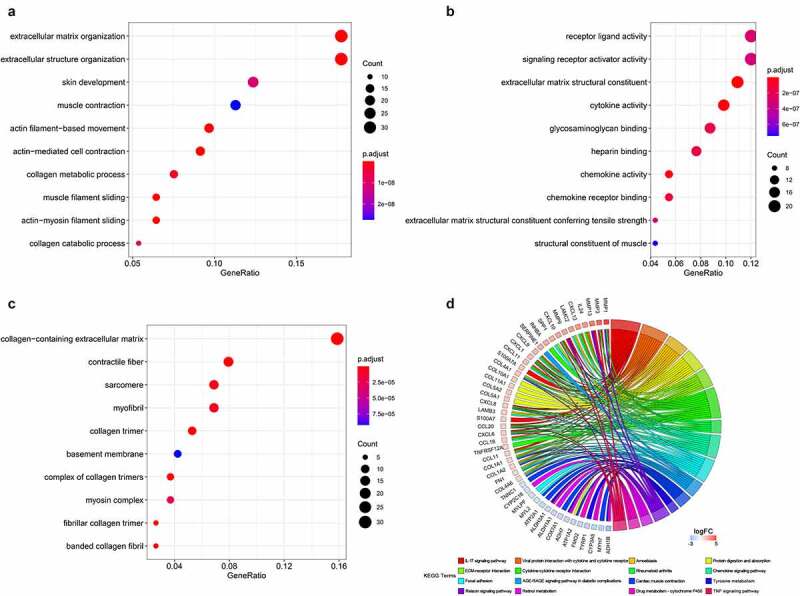
GO and KEGG annotation of the integrated DEGs. (a). Biological process. (b). Molecular function. (c). Cellular components. (d). KEGG pathways

The most significant GO items of biological process (BP) were extracellular matrix organization, extracellular structure organization, skin development, muscle contraction, and actin filament-based movement (Figure 5(a), Supplementary Table 2). The most significant molecular function (MF) items were receptor ligand activity, signaling receptor activator activity, extracellular matrix structural constituent, cytokine activity, and glycosaminoglycan binding (Figure 5(b), Supplementary Table 2). The most significant cellular component (CC) items were collagen-containing extracellular matrix, contractile fiber, sarcomeres, myofibrils, and collagen trimers (Figure 5(c), Supplementary Table 2). The most significant KEGG pathways were the IL-17 signaling pathway, viral protein interaction with cytokines and cytokine receptors, amoebiasis, protein digestion and absorption, and extracellular matrix (ECM)-receptor interaction (Figure 5(d)).

### Protein-protein interaction network

**Figure 6.**
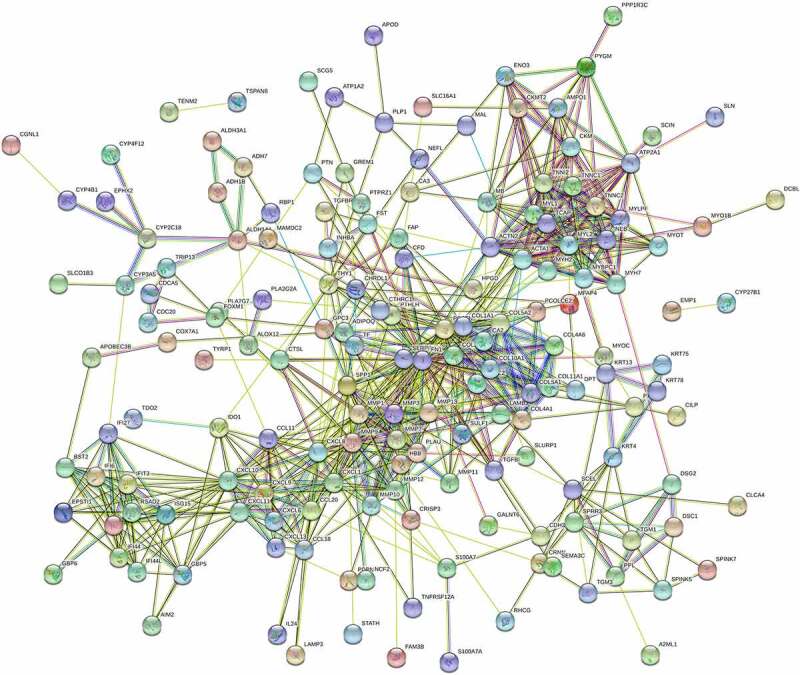
PPI meshwork of the integrated DEGs

The PPI mesh is displayed in Figure 6. There were 192 nodes and 728 edges in this PPI network. The average node degree was 7.58. In total, the p value of the PPI enrichment was less than 0.001.

### Construction of a prognostic model of OSCC based on integrated DEGs

**Figure 7.**
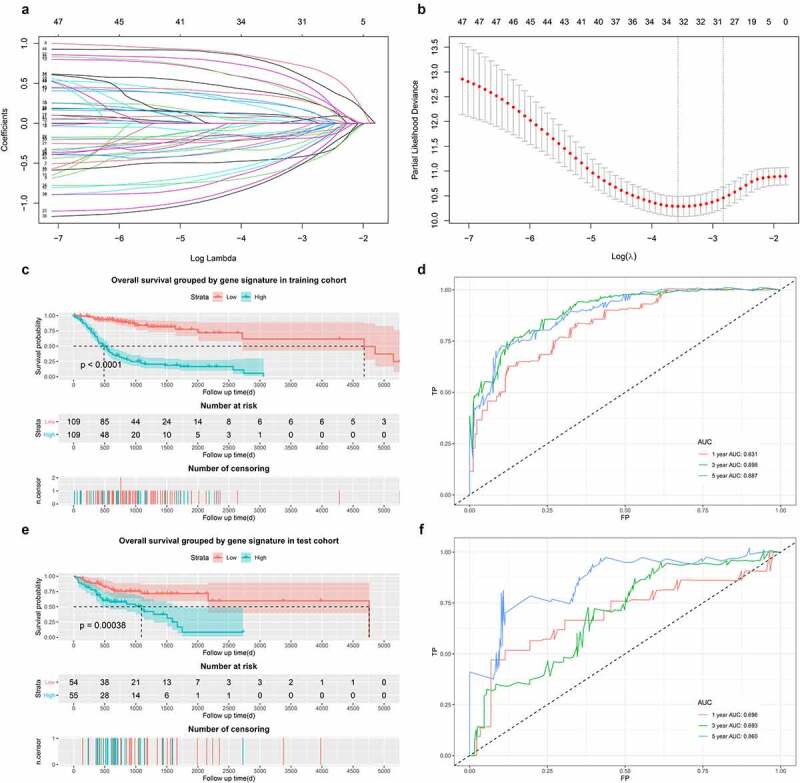
Construction of LASSO Cox model. (a) and (b). LASSO Cox model. (c). Survival curves in training cohort. (d). Time-dependent ROC curve in training cohort. (e). Survival curves in test cohort. (f). Time-dependent ROC curve in test cohort

Univariate analysis of the 193 integrated DEGs was performed, and 49 genes with p value < 0.05 were then recorded for subsequent LASSO Cox regression. A LASSO Cox regression model was built based on thirty-two genes (Figure 7(a,b)). In Figure 7(a), each adjusted parameter (λ) corresponded to a different regression model, and each smooth curve represented a parameter in the model. The ordinate of Figure 7(a) reflects the coefficients of different parameters in the model. The abscissa and ordinate of Figure 7(b) are the log λ and partial likelihood deviance, and the latter reflects the accuracy of the model. The left vertical spotted line (the minimum value of λ) was chosen, and the model acquired the highest accuracy but had more parameters. In the training cohort, the model was capable of discriminating two groups with differential overall survival (Figure 7(c), p < 0.001). The time-dependent ROC curve revealed that the AUC values at one year, three years, and five years were 0.831, 0.898, and 0.887, respectively (Figure 7(d)). In the testing cohort, patients with high gene signature scores had worse outcomes (Figure 7(e), p < 0.001). The time-dependent ROC curve showed that the AUC values at one year, three years, and five years were 0.696, 0.693, and 0.860, respectively (Figure 7(f)).

## Discussion

In our present study, integrated DEGs based on multiple microarray datasets were identified and validated by TCGA datasets in OSCC. The top five upregulated genes were MMP1, MMP10, MMP3, MMP13, and MMP12, and the top five downregulated genes were CRISP3, MAL, KRT4, TMPRSS11B, and CRNN. KEGG pathway enrichment elucidated that the integrated DEGs were enriched in the IL-17 signaling pathway, viral protein interaction with cytokines and cytokine receptors, amoebiasis, protein digestion and absorption, and ECM-receptor interaction. Furthermore, our LASSO Cox model indicated that some of the integrated DEGs could be indicators of prognosis in OSCC.

## Several mechanisms of pathogenesis in OSCC

The development of OSCC is a long and complex process, and the signaling pathways, cytokines, and regulatory mechanisms involved are still not clear [9]. Previous studies have shown that multiple dysfunctional biological processes occur during the pathogenesis of OSCC. First, OSCC is associated with severe immunosuppression; however, the surroundings of the premalignant lesion have not yet been elucidated. The growth of primary tumors is related to the presence of immune cells, which cause the common inflammatory reaction in OSCC [10]. Second, Alexander W. Eckert et al. [11] found that a hallmark of OSCC development is the constant and hypoxic stabilization of hypoxia inducible factor 1 subunit alpha (HIF1A), which is considered a general regulator in overcoming metabolic stress and energy disorders. HIF1A induces stabilization of pHi to ensure tumor cell survival and DNA replication. Third, a common discovery in the carcinogenesis of OSCC is the alteration of microRNA (miRNA) expression patterns. Mehdi Aaliet et al. [12] showed that miRNA signatures are powerful tools for the diagnosis of OSCC. For instance, a previous study [13] proved that microRNA 27b was overexpressed in saliva and expressed at low levels in plasma and OSCC tissues. Finally, epithelial to mesenchymal transition (EMT), one of the significant processes in OSCC, is described as a gradually changing process in which epithelial cells acquire some properties of mesenchymal cells, such as invasion and migration [10]. EMT is considered essential for OSCC, which endows tumors with invasion and metastasis abilities. Proteins related to EMT, such as E-cadherin, N-cadherin, and β-catenin, tend to be dysregulated in OSCC. Such disequilibrium influences the status of downstream signaling, such as the Notch, transforming growth factor β1 (TGFβ1), NF-κB, sonic hedgehog, Wnt, and epidermal growth factor receptor (EGFR) signaling pathways, which enhance the invasion and metastasis of OSCC [14]. In addition, MMPs degrade the ECM so that OSCC cells more easily infringe on surrounding tissues and metastasize to distant organs [15].

## Illustration of prognostic indicators in OSCC

Researchers are focusing on prognostic targets of OSCC. A recent study [16] proved that overexpression of HITTERS (H ERPUD1 intronic transcript of ER stress) significantly contributed to phenotypes related to cell proliferation, which indicated that HITTERS was closely related to the prognosis of OSCC. Jiayu Liang et al. [17] confirmed that solute carrier family 3 member 2 (SLC3A2) was highly expressed in OSCC. SLC3A2 could contribute to migration, invasion and proliferation and even decrease apoptosis in OSCC. Zhizhong Wu et al. [18] found that serine hydroxymethyltransferase 2 (SHMT2), which serves as an independent prognostic marker, is overexpressed in OSCC, especially in advanced OSCC. SHMT2 is implicated in neoplasia and is associated with the expression of CD274 molecule (CD274), CKLF-like MARVEL transmembrane domain containing 6 (CMTM6), V-set immunoregulatory receptor (VSIR), V-set domain containing T cell activation inhibitor 1 (VTCN1), snail family transcriptional repressor 2 (SNAI2), and bone marrow stromal cell antigen 2 (BST2) in the tumor microenvironment of OSCC.

## Elucidation of biomarkers in OSCC

Additionally, many novel biomarkers have been identified for the discrimination of OSCC and paracancerous tissue. High levels of MMP9 have been detected in the invasive tumor frontier (ITF), and many studies have applied MMP9 as a potential marker of invasive OSCC [19,20]. In OSCC, aberrant expression of circular RNAs (circRNAs) plays a role. For instance, Jingpeng Liu and coworkers [21] showed that circIGHG, an immunoglobulin heavy chain G (IGHG)-derived circRNA, is highly expressed in OSCC, which leads to the invasion and metastasis of OSCC via the microRNA (miR)-142-5p/insulin-like growth factor 2 mRNA binding protein 3 (IGF2BP3) pathway when inducing EMT. Carolina Moretto Carnielli et al. [22] applied proteomics and found that the combination of leukotriene A4 hydrolase (LTA4H)-, collagen type VI alpha 1 chain (COL6A1)-, and cystatin B (CSTB)-specific peptides in saliva, together with lymph node metastasis, can be an indicator of the prognosis of OSCC.

## Discussion of the top 10 dysregulated genes in OSCC

In our study, we identified and validated several potential biomarkers in OSCC. The top 10 OSCC-related genes were screened out (namely, MMP1, MMP10, MMP3, MMP13, MMP12, CRISP3, MAL, KRT4, TMPRSS11B and CRNN). One interesting observation is that the five members of the MMP family (MMP1, MMP10, MMP3, MMP13 and MMP12) were the most significantly upregulated genes. This result is generally consistent with the previous research results of Hui Ye et al. [23] in oral tongue squamous cell carcinoma. MMPs, enzymes that digest components of the ECM [24], are secreted by macrophages, neutrophils, and fibroblasts stimulated by transforming growth factor β (TGF-β) and interleukin 8 (IL-8).

Among the MMPs, MMP1 is the most commonly expressed interstitial collagenase and breaks down ECM via the cleavage of type I, II and III collagens. The expression of MMP1 was higher in primary OSCC than in normal oral epithelium [25]. A recent study also found a higher level of MMP1 in OSCC patients with advanced clinical stages and grades [26]. Furthermore, salivary MMP1 might be a useful biomarker for monitoring malignant transformation from oral potentially malignant disorders to OSCC. MMP10 breaks down fibronectin, laminin-5, elastin, proteoglycan core protein, gelatins, and types III–V collagen, among others [27]. Elsayed Mohamed Deraz et al. [28] previously demonstrated that high expression of MMP10 could contribute to the invasion and metastasis of HNSCC, and the invasion induced by MMP10 is related to p38 mitogen-activated protein kinase (MAPK) inhibition. MMP3 is a kind of stromelysin and breaks down many extracellular matrix components, such as proteoglycans, fibronectin, laminin and collagen types III, IV, and V. Yu Jin et al. [29] reported that a high level of MMP3 expression in OSCC tissues was also correlated with tumor metastasis and poor prognosis of OSCC patients. MMP13 preferably breaks down type II collagen [30]. Zhenhu Ren et al. [31] demonstrated that MMP13 levels were higher in OSCC patients than in healthy individuals. MMP13 could serve as an indicator of metastasis and invasion in OSCC. MMP12 has been found to have significant sensitivity and specificity to qualify as a diagnostic biomarker in OSCC [32].

As a member of the cysteine-rich secretory protein (CRISP) family, CRISP3 is expressed in a biased manner in the salivary glands, bone marrow, and esophagus. Previous reports clearly indicate [33,34] that the expression of CRISP3 in prostate carcinoma and mammary carcinoma is obviously upregulated. Marianna Volpert et al. [33] found that CRISP3 expression drives the invasion and progression of prostate cancer. A previous study [34] indicated that CRISP3 was involved in the invasion and migration of mammary cancer and that CRISP3 was closely correlated with poor prognosis in mammary cancer patients. However, the role of CRISP3 in OSCC is not well known. In a study by WEN-CHANG KO et al. [35], the results suggested that CRISP3 was a novel tumor suppressor gene specific to OSCC, and inactivation of CRISP3 may be involved in the carcinogenesis of OSCC patients. MAL, a highly hydrophobic integral membrane protein of proteolipids, is a differentially expressed gene closely related to the development of T-cells. Samir Kumar Pal et al. [36] found that the expression of MAL was significantly lower in OSCC than in normal oral epithelium. KRT4 belongs to the keratin gene family and is clustered in a region of chromosome 12q12-q13. KRT4 was previously reported to be a downregulated gene in OSCC compared with normal tissues [37]. Another study also demonstrated [3] that downregulated expression of KRT4 is a fundamental feature observed in OSCC [38]. CRNN is a part of the ”fused gene” family of proteins located on the chromosome 1q21 locus. It may be involved in the immune response and differentiation of the epidermis. Previous studies have reported that CRNN is downregulated in OSCC and esophageal squamous cell carcinoma [39,40]. Additionally, low expression of CRNN was associated with poor prognosis in OSCC [41].

## Conclusion

The differentially expressed genes of OSCC were associated with potential pathways related to pathogenesis, and these genes were applicable in the evaluation of prognosis in OSCC. However, further research should be performed to validate our findings.

## Supplementary Material

Supplemental Material

## Data Availability

The datasets analyzed was acquired from The Cancer Genome Atlas (TCGA) database (https://portal.gdc.cancer.gov/) and GEO database (https://www.ncbi.nlm.nih.gov/geo/).

## Highlights

1. MMP1, MMP10, MMP3, MMP13, and MMP12 were the most highly upregulated genes in OSCC.
2. CRISP3, MAL, KRT4, TMPRSS11B, and CRNN were the most highly downregulated genes in OSCC.
3. The differentially expressed genes of OSCC might be applicable in the evaluation of prognosis in OSCC.
