# Supplementary material for: The integration of differentially expressed genes based on multiple microarray datasets for prediction of the prognosis in oral squamous cell carcinoma
Source: Bioengineered. 2021 Jul 5;12(1):3309–21. doi: 10.1080/21655979.2021.1947076 (PMC8806768; doi:10.1080/21655979.2021.1947076)
Supplement: Supplemental Material [file KBIE_A_1947076_SM9320.zip › supplementary/S Table 1.docx]

Table 1. Characteristics of the included datasets of oral squamous cells carcinoma.

| GEO accession | Year | Country or region | Cases number | Control number | Platform |
| --- | --- | --- | --- | --- | --- |
| GSE138206 | 2019 | China | 6 | 12 | GPL570 |
| GSE107591 | 2017 | Italy | 17 | 16 | GPL6244 |
| GSE74530 | 2017 | USA | 6 | 6 | GPL570 |
| GSE75538 | 2016 | India | 14 | 14 | GPL18281 |
| GSE37991 | 2013 | Taiwan | 40 | 40 | GPL6883 |
| GSE35261 | 2012 | Japan | 11 | 22 | GPL8950 |
| GSE31056 | 2011 | USA | 23 | 73 | GPL10526 |
| GSE30784 | 2011 | USA | 167 | 62 | GPL570 |
| GSE13601 | 2008 | USA | 31 | 26 | GPL8300 |
| GSE9844 | 2008 | USA | 26 | 12 | GPL570 |
