# Supplementary material for: The integration of differentially expressed genes based on multiple microarray datasets for prediction of the prognosis in oral squamous cell carcinoma
Source: Bioengineered. 2021 Jul 5;12(1):3309–21. doi: 10.1080/21655979.2021.1947076 (PMC8806768; doi:10.1080/21655979.2021.1947076)
Supplement: Supplemental Material [file KBIE_A_1947076_SM9320.zip › supplementary/S Table 2.docx]

Supplementary Table 2. Enrichment GO results based on the integrated DEGs.

| Category | ID | Description | geneID | Count |
| --- | --- | --- | --- | --- |
| BP | GO:0030198 | extracellular matrix organization | MMP1/MMP10/MMP3/MMP13/MMP12/MMP9/LAMC2/SERPINE1/SPP1/MMP7/FAP/COL5A2/POSTN/LAMB3/COL5A1/FN1/TGFBI/GREM1/COL4A1/PDPN/MMP11/COL10A1/COL1A1/COL11A1/PXDN/SULF1/COL1A2/CTSL/COL4A6/SPINK5/DPT/MFAP4/VIT | 33 |
| BP | GO:0043062 | extracellular structure organization | MMP1/MMP10/MMP3/MMP13/MMP12/MMP9/LAMC2/SERPINE1/SPP1/MMP7/FAP/COL5A2/POSTN/LAMB3/COL5A1/FN1/TGFBI/GREM1/COL4A1/PDPN/MMP11/COL10A1/COL1A1/COL11A1/PXDN/SULF1/COL1A2/CTSL/COL4A6/SPINK5/DPT/MFAP4/VIT | 33 |
| BP | GO:0030049 | muscle filament sliding | MYH2/ACTA1/MYBPC1/MYH7/MYL1/TNNI2/MYL2/TNNC2/TNNC1/ACTN2/TCAP/NEB | 12 |
| BP | GO:0033275 | actin-myosin filament sliding | MYH2/ACTA1/MYBPC1/MYH7/MYL1/TNNI2/MYL2/TNNC2/TNNC1/ACTN2/TCAP/NEB | 12 |
| BP | GO:0070252 | actin-mediated cell contraction | PDPN/DSG2/MYH2/ATP1A2/GPD1L/ACTA1/MYBPC1/MYH7/MYL1/TNNI2/MYL2/TNNC2/TNNC1/ATP2A1/ACTN2/TCAP/NEB | 17 |
| BP | GO:0030048 | actin filament-based movement | PDPN/MYO1B/DSG2/MYH2/ATP1A2/GPD1L/ACTA1/MYBPC1/MYH7/MYL1/TNNI2/MYL2/TNNC2/TNNC1/ATP2A1/ACTN2/TCAP/NEB | 18 |
| BP | GO:0032963 | collagen metabolic process | MMP1/MMP10/MMP3/MMP13/MMP12/MMP9/MMP7/FAP/COL5A1/MMP11/COL1A1/COL1A2/CTSL/MFAP4 | 14 |
| BP | GO:0030574 | collagen catabolic process | MMP1/MMP10/MMP3/MMP13/MMP12/MMP9/MMP7/FAP/MMP11/CTSL | 10 |
| BP | GO:0043588 | skin development | CDH3/INHBA/COL5A2/DSC1/S100A7/COL5A1/KRT75/FST/COL1A1/CYP27B1/COL1A2/CTSL/DSG2/KRT4/KRT13/KRT78/TGM3/SPINK5/PPL/ALOX12/SPRR3/SCEL/TGM1 | 23 |
| BP | GO:0006936 | muscle contraction | SULF1/DSG2/MYH2/ATP1A2/GPD1L/ACTA1/MYBPC1/MB/MYH7/MYL1/TNNI2/MYL2/TNNC2/MYOT/TNNC1/ATP2A1/ACTN2/CKMT2/TCAP/MYLPF/NEB | 21 |
| BP | GO:0003012 | muscle system process | SULF1/DSG2/MYH2/ATP1A2/GPD1L/ACTA1/MYBPC1/MB/MYH7/MYL1/SLN/TNNI2/MYL2/TNNC2/MYOT/MYOC/TNNC1/ATP2A1/ACTN2/CKMT2/TCAP/MYLPF/NEB | 23 |
| BP | GO:0022617 | extracellular matrix disassembly | MMP1/MMP10/MMP3/MMP13/MMP12/MMP9/MMP7/FAP/PDPN/MMP11/CTSL | 11 |
| BP | GO:0008544 | epidermis development | CDH3/PTHLH/LAMC2/INHBA/DSC1/S100A7/LAMB3/KRT75/FST/CYP27B1/CTSL/DSG2/KRT4/KRT13/KRT78/TGM3/SPINK5/PPL/SPRR3/SCEL/EMP1/TGM1 | 22 |
| BP | GO:0030199 | collagen fibril organization | COL5A2/COL5A1/GREM1/MMP11/COL1A1/COL11A1/PXDN/COL1A2/DPT | 9 |
| BP | GO:0050900 | leukocyte migration | MMP1/CXCL13/CXCL10/SERPINE1/CXCL9/CXCL11/PLA2G7/S100A7/FN1/GREM1/THY1/SLC16A1/CXCL8/CXCL1/COL1A1/CCL20/CCL18/CCL11/COL1A2/CXCL6/PTN/APOD | 22 |
| BP | GO:0030595 | leukocyte chemotaxis | CXCL13/CXCL10/SERPINE1/CXCL9/CXCL11/PLA2G7/S100A7/GREM1/CXCL8/CXCL1/CCL20/CCL18/CCL11/CXCL6/PTN | 15 |
| BP | GO:0070098 | chemokine-mediated signaling pathway | CXCL13/CXCL10/CXCL9/CXCL11/CXCL8/CXCL1/CCL20/CCL18/CCL11/CXCL6 | 10 |
| BP | GO:0097529 | myeloid leukocyte migration | CXCL13/CXCL10/SERPINE1/CXCL9/CXCL11/PLA2G7/S100A7/GREM1/CXCL8/CXCL1/CCL20/CCL18/CCL11/CXCL6 | 14 |
| BP | GO:0071621 | granulocyte chemotaxis | CXCL13/CXCL10/CXCL9/CXCL11/S100A7/CXCL8/CXCL1/CCL20/CCL18/CCL11/CXCL6 | 11 |
| BP | GO:0061844 | antimicrobial humoral immune response mediated by antimicrobial peptide | CXCL13/CXCL10/CXCL9/CXCL11/S100A7/CXCL8/CXCL1/CXCL6/SPINK5 | 9 |
| BP | GO:1990868 | response to chemokine | CXCL13/CXCL10/CXCL9/CXCL11/CXCL8/CXCL1/CCL20/CCL18/CCL11/CXCL6 | 10 |
| BP | GO:1990869 | cellular response to chemokine | CXCL13/CXCL10/CXCL9/CXCL11/CXCL8/CXCL1/CCL20/CCL18/CCL11/CXCL6 | 10 |
| BP | GO:0030216 | keratinocyte differentiation | CDH3/DSC1/S100A7/KRT75/CYP27B1/CTSL/DSG2/KRT4/KRT13/KRT78/TGM3/SPINK5/PPL/SPRR3/SCEL/TGM1 | 16 |
| BP | GO:0030593 | neutrophil chemotaxis | CXCL13/CXCL10/CXCL9/CXCL11/CXCL8/CXCL1/CCL20/CCL18/CCL11/CXCL6 | 10 |
| BP | GO:0009615 | response to virus | MMP12/CXCL10/RSAD2/ISG15/IFI6/IFIT3/CXCL9/AIM2/BST2/OASL/IFI27/CCL11/GSDME/IFI44/IFI44L/APOBEC3B | 16 |
| BP | GO:0097530 | granulocyte migration | CXCL13/CXCL10/CXCL9/CXCL11/S100A7/CXCL8/CXCL1/CCL20/CCL18/CCL11/CXCL6 | 11 |
| BP | GO:0070268 | cornification | DSC1/KRT75/DSG2/KRT4/KRT13/KRT78/SPINK5/PPL/SPRR3/TGM1 | 10 |
| BP | GO:0003009 | skeletal muscle contraction | MB/MYH7/TNNI2/TNNC2/TNNC1/ATP2A1/TCAP | 7 |
| BP | GO:0006941 | striated muscle contraction | DSG2/ATP1A2/GPD1L/MB/MYH7/MYL1/TNNI2/MYL2/TNNC2/TNNC1/ATP2A1/TCAP | 12 |
| BP | GO:0002548 | monocyte chemotaxis | CXCL10/SERPINE1/PLA2G7/S100A7/GREM1/CCL20/CCL18/CCL11 | 8 |
| BP | GO:0035987 | endodermal cell differentiation | MMP9/INHBA/COL5A2/LAMB3/COL5A1/FN1/COL11A1 | 7 |
| BP | GO:1990266 | neutrophil migration | CXCL13/CXCL10/CXCL9/CXCL11/CXCL8/CXCL1/CCL20/CCL18/CCL11/CXCL6 | 10 |
| BP | GO:0019730 | antimicrobial humoral response | CXCL13/CXCL10/CXCL9/CXCL11/S100A7/CXCL8/CXCL1/CXCL6/SPINK5/PLA2G2A | 10 |
| BP | GO:0060326 | cell chemotaxis | CXCL13/CXCL10/SERPINE1/CXCL9/CXCL11/PLA2G7/S100A7/GREM1/CXCL8/CXCL1/CCL20/CCL18/CCL11/CXCL6/PTN | 15 |
| BP | GO:0031589 | cell-substrate adhesion | MMP12/LAMC2/PLAU/SERPINE1/POSTN/LAMB3/FN1/THY1/PDPN/COL1A1/TNFRSF12A/PTN/APOD/MYOC/ACTN2/VIT | 16 |
| BP | GO:0001706 | endoderm formation | MMP9/INHBA/COL5A2/LAMB3/COL5A1/FN1/COL11A1 | 7 |
| BP | GO:0009913 | epidermal cell differentiation | CDH3/DSC1/S100A7/KRT75/CYP27B1/CTSL/DSG2/KRT4/KRT13/KRT78/TGM3/SPINK5/PPL/SPRR3/SCEL/TGM1 | 16 |
| BP | GO:0031214 | biomineral tissue development | MMP13/PTHLH/ISG15/SPP1/GREM1/COL1A1/CYP27B1/COL1A2/PTN/STATH/GPC3 | 11 |
| BP | GO:0110148 | biomineralization | MMP13/PTHLH/ISG15/SPP1/GREM1/COL1A1/CYP27B1/COL1A2/PTN/STATH/GPC3 | 11 |
| BP | GO:0051607 | defense response to virus | MMP12/CXCL10/RSAD2/ISG15/IFI6/IFIT3/CXCL9/AIM2/BST2/OASL/IFI27/IFI44L/APOBEC3B | 13 |
| BP | GO:0007492 | endoderm development | MMP9/INHBA/COL5A2/LAMB3/COL5A1/FN1/COL11A1/PAX9 | 8 |
| BP | GO:0050879 | multicellular organismal movement | MB/MYH7/TNNI2/TNNC2/TNNC1/ATP2A1/TCAP | 7 |
| BP | GO:0050881 | musculoskeletal movement | MB/MYH7/TNNI2/TNNC2/TNNC1/ATP2A1/TCAP | 7 |
| BP | GO:0060048 | cardiac muscle contraction | DSG2/ATP1A2/GPD1L/MYH7/MYL1/TNNI2/MYL2/TNNC1/ATP2A1/TCAP | 10 |
| BP | GO:0010810 | regulation of cell-substrate adhesion | MMP12/PLAU/SERPINE1/POSTN/FN1/THY1/PDPN/COL1A1/PTN/APOD/MYOC/VIT | 12 |
| BP | GO:0030282 | bone mineralization | MMP13/PTHLH/ISG15/GREM1/CYP27B1/COL1A2/PTN/STATH/GPC3 | 9 |
| BP | GO:0001503 | ossification | MMP13/PTHLH/ISG15/SPP1/COL5A2/CTHRC1/GREM1/COL1A1/CYP27B1/COL11A1/COL1A2/CHRDL1/PTN/MYOC/STATH/GPC3 | 16 |
| BP | GO:0031424 | keratinization | CDH3/DSC1/KRT75/DSG2/KRT4/KRT13/KRT78/TGM3/SPINK5/PPL/SPRR3/TGM1 | 12 |
| BP | GO:0071674 | mononuclear cell migration | CXCL10/SERPINE1/PLA2G7/S100A7/GREM1/CCL20/CCL18/CCL11 | 8 |
| BP | GO:0048247 | lymphocyte chemotaxis | CXCL13/CXCL10/CXCL11/S100A7/CCL20/CCL18/CCL11 | 7 |
| BP | GO:0090025 | regulation of monocyte chemotaxis | CXCL10/SERPINE1/PLA2G7/S100A7/GREM1 | 5 |
| BP | GO:0002685 | regulation of leukocyte migration | CXCL13/CXCL10/SERPINE1/PLA2G7/S100A7/GREM1/THY1/CXCL8/CCL20/PTN/APOD | 11 |
| BP | GO:0060337 | type I interferon signaling pathway | MMP12/RSAD2/ISG15/IFI6/IFIT3/BST2/OASL/IFI27 | 8 |
| BP | GO:0071357 | cellular response to type I interferon | MMP12/RSAD2/ISG15/IFI6/IFIT3/BST2/OASL/IFI27 | 8 |
| BP | GO:0002687 | positive regulation of leukocyte migration | CXCL13/CXCL10/SERPINE1/PLA2G7/S100A7/THY1/CXCL8/CCL20/PTN | 9 |
| BP | GO:0055008 | cardiac muscle tissue morphogenesis | COL11A1/TGFBR3/MYBPC1/MYH7/MYL2/TNNC1/TCAP | 7 |
| BP | GO:0034340 | response to type I interferon | MMP12/RSAD2/ISG15/IFI6/IFIT3/BST2/OASL/IFI27 | 8 |
| BP | GO:0009612 | response to mechanical stimulus | CXCL10/MMP7/POSTN/COL1A1/COL11A1/ATP1A2/ACTA1/PTN/PPL/SCEL/TCAP | 11 |
| BP | GO:0014706 | striated muscle tissue development | SEMA3C/GREM1/COL11A1/DSG2/HLF/TGFBR3/ACTA1/MYBPC1/MYH7/MYL2/TNNC1/ACTN2/TCAP/MYLPF/NEB | 15 |
| BP | GO:0042573 | retinoic acid metabolic process | RBP1/ADH1B/CYP3A5/ADH7/CYP2C18 | 5 |
| BP | GO:0060537 | muscle tissue development | SEMA3C/GREM1/COL11A1/DSG2/HLF/TGFBR3/ACTA1/MYBPC1/MYH7/MYL2/TNNC1/ACTN2/TCAP/MYLPF/NEB | 15 |
| BP | GO:0072676 | lymphocyte migration | CXCL13/CXCL10/CXCL11/S100A7/CCL20/CCL18/CCL11/APOD | 8 |
| BP | GO:0003229 | ventricular cardiac muscle tissue development | COL11A1/DSG2/TGFBR3/MYH7/MYL2/TNNC1 | 6 |
| BP | GO:0060415 | muscle tissue morphogenesis | COL11A1/TGFBR3/MYBPC1/MYH7/MYL2/TNNC1/TCAP | 7 |
| BP | GO:0002688 | regulation of leukocyte chemotaxis | CXCL13/CXCL10/SERPINE1/PLA2G7/S100A7/GREM1/CXCL8/PTN | 8 |
| BP | GO:0048738 | cardiac muscle tissue development | GREM1/COL11A1/DSG2/TGFBR3/MYBPC1/MYH7/MYL2/TNNC1/ACTN2/TCAP/NEB | 11 |
| BP | GO:0007565 | female pregnancy | MMP9/PTHLH/SPP1/MMP7/IDO1/CYP27B1/DSG2/PTN/HPGD/ENDOU | 10 |
| BP | GO:0002690 | positive regulation of leukocyte chemotaxis | CXCL13/CXCL10/SERPINE1/PLA2G7/S100A7/CXCL8/PTN | 7 |
| BP | GO:0090026 | positive regulation of monocyte chemotaxis | CXCL10/SERPINE1/PLA2G7/S100A7 | 4 |
| BP | GO:0032496 | response to lipopolysaccharide | IL24/CXCL13/CXCL10/SERPINE1/CXCL9/CXCL11/S100A7/CXCL8/CXCL1/IDO1/CYP27B1/CXCL6/HPGD | 13 |
| BP | GO:0048644 | muscle organ morphogenesis | COL11A1/TGFBR3/MYBPC1/MYH7/MYL2/TNNC1/TCAP | 7 |
| BP | GO:0016999 | antibiotic metabolic process | MMP3/PXDN/ADH1B/CYP4B1/ALDH1A1/GPX3/ADH7/HBB | 8 |
| BP | GO:0002237 | response to molecule of bacterial origin | IL24/CXCL13/CXCL10/SERPINE1/CXCL9/CXCL11/S100A7/CXCL8/CXCL1/IDO1/CYP27B1/CXCL6/HPGD | 13 |
| BP | GO:0072678 | T cell migration | CXCL13/CXCL10/CXCL11/S100A7/CCL20/APOD | 6 |
| BP | GO:0007044 | cell-substrate junction assembly | LAMC2/LAMB3/FN1/THY1/APOD/MYOC/ACTN2 | 7 |
| BP | GO:0150115 | cell-substrate junction organization | LAMC2/LAMB3/FN1/THY1/APOD/MYOC/ACTN2 | 7 |
| BP | GO:0010812 | negative regulation of cell-substrate adhesion | MMP12/SERPINE1/POSTN/COL1A1/APOD/MYOC | 6 |
| BP | GO:0001953 | negative regulation of cell-matrix adhesion | MMP12/SERPINE1/POSTN/APOD/MYOC | 5 |
| BP | GO:2000404 | regulation of T cell migration | CXCL13/CXCL10/S100A7/CCL20/APOD | 5 |
| BP | GO:1903034 | regulation of response to wounding | PLAU/SERPINE1/SPP1/FAP/PDPN/TNFRSF12A/PTN/TSPAN8/ALOX12 | 9 |
| BP | GO:0044706 | multi-multicellular organism process | MMP9/PTHLH/SPP1/MMP7/IDO1/CYP27B1/DSG2/PTN/HPGD/ENDOU | 10 |
| BP | GO:0001523 | retinoid metabolic process | RBP1/ADH1B/CYP3A5/ALDH1A1/ADH7/GPC3/CYP2C18 | 7 |
| BP | GO:0071677 | positive regulation of mononuclear cell migration | CXCL10/SERPINE1/PLA2G7/S100A7 | 4 |
| BP | GO:0003208 | cardiac ventricle morphogenesis | SEMA3C/COL11A1/TGFBR3/MYH7/MYL2/TNNC1 | 6 |
| BP | GO:0030239 | myofibril assembly | ACTA1/MYBPC1/MYL2/ACTN2/TCAP/NEB | 6 |
| BP | GO:0071675 | regulation of mononuclear cell migration | CXCL10/SERPINE1/PLA2G7/S100A7/GREM1 | 5 |
| BP | GO:1903115 | regulation of actin filament-based movement | PDPN/DSG2/ATP1A2/TNNC1/ATP2A1 | 5 |
| BP | GO:0007369 | gastrulation | MMP9/INHBA/COL5A2/LAMB3/COL5A1/FN1/COL11A1/ADIPOQ/GPC3 | 9 |
| BP | GO:0038063 | collagen-activated tyrosine kinase receptor signaling pathway | COL4A1/COL1A1/COL4A6 | 3 |
| BP | GO:0055010 | ventricular cardiac muscle tissue morphogenesis | COL11A1/TGFBR3/MYH7/MYL2/TNNC1 | 5 |
| BP | GO:0016101 | diterpenoid metabolic process | RBP1/ADH1B/CYP3A5/ALDH1A1/ADH7/GPC3/CYP2C18 | 7 |
| BP | GO:0055003 | cardiac myofibril assembly | MYBPC1/MYL2/TCAP/NEB | 4 |
| BP | GO:0071711 | basement membrane organization | LAMB3/COL4A1/MMP11/PXDN | 4 |
| BP | GO:0051271 | negative regulation of cellular component movement | IL24/CXCL13/SERPINE1/BST2/SEMA3C/GREM1/THY1/SULF1/TGFBR3/PTN/APOD/SLURP1/ADIPOQ | 13 |
| BP | GO:0030193 | regulation of blood coagulation | PLAU/SERPINE1/FAP/PDPN/TSPAN8/ALOX12 | 6 |
| BP | GO:1900046 | regulation of hemostasis | PLAU/SERPINE1/FAP/PDPN/TSPAN8/ALOX12 | 6 |
| BP | GO:0010818 | T cell chemotaxis | CXCL13/CXCL10/CXCL11/S100A7 | 4 |
| BP | GO:0043501 | skeletal muscle adaptation | ACTA1/MYH7/MYOC/TNNC1 | 4 |
| BP | GO:0030195 | negative regulation of blood coagulation | PLAU/SERPINE1/FAP/TSPAN8/ALOX12 | 5 |
| BP | GO:0001952 | regulation of cell-matrix adhesion | MMP12/PLAU/SERPINE1/POSTN/THY1/APOD/MYOC | 7 |
| BP | GO:0050818 | regulation of coagulation | PLAU/SERPINE1/FAP/PDPN/TSPAN8/ALOX12 | 6 |
| BP | GO:0032102 | negative regulation of response to external stimulus | MMP12/CXCL13/PLAU/SERPINE1/SPP1/FAP/SEMA3C/GREM1/SPINK5/APOD/TSPAN8/ADIPOQ/ALOX12 | 13 |
| BP | GO:1900047 | negative regulation of hemostasis | PLAU/SERPINE1/FAP/TSPAN8/ALOX12 | 5 |
| BP | GO:0006069 | ethanol oxidation | ADH1B/ALDH1A1/ADH7 | 3 |
| BP | GO:0006721 | terpenoid metabolic process | RBP1/ADH1B/CYP3A5/ALDH1A1/ADH7/GPC3/CYP2C18 | 7 |
| BP | GO:0007178 | transmembrane receptor protein serine/threonine kinase signaling pathway | INHBA/GREM1/PMEPA1/FST/SULF1/COL1A2/TGFBR3/CHRDL1/CILP/HPGD/TMEM100/GPC3 | 12 |
| BP | GO:0001704 | formation of primary germ layer | MMP9/INHBA/COL5A2/LAMB3/COL5A1/FN1/COL11A1 | 7 |
| BP | GO:0034764 | positive regulation of transmembrane transport | CXCL10/CXCL9/CXCL11/THY1/CA2/ADIPOQ/ATP2A1/ACTN2/GPC3 | 9 |
| BP | GO:0042759 | long-chain fatty acid biosynthetic process | PLP1/ALOX12/HPGD/EPHX2 | 4 |
| BP | GO:0071222 | cellular response to lipopolysaccharide | IL24/CXCL13/CXCL10/SERPINE1/CXCL9/CXCL11/CXCL8/CXCL1/CXCL6 | 9 |
| BP | GO:0002062 | chondrocyte differentiation | PTHLH/TGFBI/GREM1/COL11A1/SULF1/SCIN/VIT | 7 |
| BP | GO:2000406 | positive regulation of T cell migration | CXCL13/CXCL10/S100A7/CCL20 | 4 |
| BP | GO:0007517 | muscle organ development | CXCL10/GREM1/COL11A1/HLF/TGFBR3/ACTA1/MYBPC1/MYH7/MYL2/TNNC1/TCAP/MYLPF/NEB | 13 |
| BP | GO:0051216 | cartilage development | MMP13/PTHLH/TGFBI/GREM1/COL1A1/COL11A1/SULF1/SCIN/VIT | 9 |
| BP | GO:0014888 | striated muscle adaptation | ACTA1/MYH7/MYOC/TNNC1/TCAP | 5 |
| BP | GO:0050819 | negative regulation of coagulation | PLAU/SERPINE1/FAP/TSPAN8/ALOX12 | 5 |
| BP | GO:0038065 | collagen-activated signaling pathway | COL4A1/COL1A1/COL4A6 | 3 |
| BP | GO:0070208 | protein heterotrimerization | COL1A1/COL1A2/ADIPOQ | 3 |
| BP | GO:0035051 | cardiocyte differentiation | SEMA3C/GREM1/TGFBR3/MYBPC1/MYL2/ACTN2/TCAP/NEB | 8 |
| BP | GO:0071295 | cellular response to vitamin | POSTN/COL1A1/CYP27B1/PTN | 4 |
| BP | GO:0003007 | heart morphogenesis | COL5A1/SEMA3C/COL11A1/TGFBR3/MYBPC1/MYH7/MYL2/TNNC1/TMEM100/TCAP | 10 |
| BP | GO:1903035 | negative regulation of response to wounding | PLAU/SERPINE1/SPP1/FAP/TSPAN8/ALOX12 | 6 |
| BP | GO:0071219 | cellular response to molecule of bacterial origin | IL24/CXCL13/CXCL10/SERPINE1/CXCL9/CXCL11/CXCL8/CXCL1/CXCL6 | 9 |
| BP | GO:0031102 | neuron projection regeneration | SPP1/THY1/NEFL/PTN/APOD | 5 |
| BP | GO:0034754 | cellular hormone metabolic process | SPP1/RBP1/ADH1B/CYP3A5/ALDH1A1/ADH7/CYP2C18 | 7 |
| BP | GO:0006937 | regulation of muscle contraction | DSG2/ATP1A2/MYH7/TNNI2/MYL2/TNNC2/TNNC1/ATP2A1 | 8 |
| BP | GO:0030240 | skeletal muscle thin filament assembly | ACTA1/MYBPC1/TCAP | 3 |
| BP | GO:0033273 | response to vitamin | CXCL10/SPP1/POSTN/COL1A1/CYP27B1/PTN | 6 |
| BP | GO:0050920 | regulation of chemotaxis | CXCL13/CXCL10/SERPINE1/PLA2G7/S100A7/SEMA3C/GREM1/CXCL8/PTN | 9 |
| BP | GO:0007584 | response to nutrient | CXCL10/SPP1/POSTN/SLC16A1/COL1A1/CYP27B1/PTN/ALDH3A1/ADIPOQ | 9 |
| BP | GO:0045069 | regulation of viral genome replication | RSAD2/ISG15/BST2/OASL/IFI27/CXCL8 | 6 |
| BP | GO:0055006 | cardiac cell development | TGFBR3/MYBPC1/MYL2/ACTN2/TCAP/NEB | 6 |
| BP | GO:2000401 | regulation of lymphocyte migration | CXCL13/CXCL10/S100A7/CCL20/APOD | 5 |
| BP | GO:0050921 | positive regulation of chemotaxis | CXCL13/CXCL10/SERPINE1/PLA2G7/S100A7/CXCL8/PTN | 7 |
| BP | GO:0061448 | connective tissue development | MMP13/PTHLH/COL5A1/TGFBI/GREM1/COL1A1/COL11A1/SULF1/SCIN/VIT | 10 |
| BP | GO:0007160 | cell-matrix adhesion | MMP12/PLAU/SERPINE1/POSTN/FN1/THY1/APOD/MYOC/ACTN2 | 9 |
| BP | GO:0060135 | maternal process involved in female pregnancy | SPP1/MMP7/CYP27B1/DSG2/PTN | 5 |
| BP | GO:0040013 | negative regulation of locomotion | IL24/CXCL13/SERPINE1/BST2/SEMA3C/GREM1/THY1/SULF1/PTN/APOD/SLURP1/ADIPOQ | 12 |
| BP | GO:0033280 | response to vitamin D | CXCL10/SPP1/CYP27B1/PTN | 4 |
| BP | GO:2000403 | positive regulation of lymphocyte migration | CXCL13/CXCL10/S100A7/CCL20 | 4 |
| BP | GO:0006720 | isoprenoid metabolic process | RBP1/ADH1B/CYP3A5/ALDH1A1/ADH7/GPC3/CYP2C18 | 7 |
| BP | GO:0034446 | substrate adhesion-dependent cell spreading | LAMC2/POSTN/LAMB3/FN1/PDPN/MYOC | 6 |
| BP | GO:0014866 | skeletal myofibril assembly | ACTA1/MYBPC1/TCAP | 3 |
| BP | GO:0042737 | drug catabolic process | PXDN/CYP3A5/CYP4B1/GPX3/ADH7/HBB/CYP2C18 | 7 |
| BP | GO:0030336 | negative regulation of cell migration | IL24/CXCL13/SERPINE1/BST2/GREM1/THY1/SULF1/PTN/APOD/SLURP1/ADIPOQ | 11 |
| BP | GO:0048747 | muscle fiber development | ACTA1/MYBPC1/MYL2/TCAP/NEB | 5 |
| BP | GO:0060047 | heart contraction | DSG2/ATP1A2/GPD1L/MYH7/MYL1/TNNI2/MYL2/TNNC1/ATP2A1/TCAP | 10 |
| BP | GO:0042445 | hormone metabolic process | SPP1/RBP1/SCG5/CYP27B1/ADH1B/CYP3A5/ALDH1A1/ADH7/CYP2C18 | 9 |
| BP | GO:0071230 | cellular response to amino acid stimulus | COL5A2/COL4A1/COL1A1/COL1A2/RRAGD | 5 |
| BP | GO:0010819 | regulation of T cell chemotaxis | CXCL13/CXCL10/S100A7 | 3 |
| BP | GO:0071216 | cellular response to biotic stimulus | IL24/CXCL13/CXCL10/SERPINE1/CXCL9/CXCL11/CXCL8/CXCL1/CXCL6 | 9 |
| BP | GO:0052547 | regulation of peptidase activity | MMP9/SERPINE1/IFI6/AIM2/BST2/FN1/LAMP3/SPINK5/SPINK7/ALOX12/PCOLCE2/A2ML1/GPC3 | 13 |
| BP | GO:1904062 | regulation of cation transmembrane transport | MMP9/CXCL10/CXCL9/CXCL11/THY1/NEFL/ATP1A2/GPD1L/SLN/ATP2A1/ACTN2 | 11 |
| BP | GO:0051281 | positive regulation of release of sequestered calcium ion into cytosol | CXCL10/CXCL9/CXCL11/THY1 | 4 |
| BP | GO:0007162 | negative regulation of cell adhesion | MMP12/SERPINE1/POSTN/TGFBI/COL1A1/IDO1/APOD/ADIPOQ/ALOX12/MYOC | 10 |
| BP | GO:0071356 | cellular response to tumor necrosis factor | AIM2/POSTN/CXCL8/COL1A1/CCL20/CCL18/CCL11/GSDME/TNFRSF12A/ADIPOQ | 10 |
| BP | GO:1904427 | positive regulation of calcium ion transmembrane transport | CXCL10/CXCL9/CXCL11/THY1/ATP2A1 | 5 |
| BP | GO:0061041 | regulation of wound healing | PLAU/SERPINE1/FAP/PDPN/TNFRSF12A/TSPAN8/ALOX12 | 7 |
| BP | GO:0003015 | heart process | DSG2/ATP1A2/GPD1L/MYH7/MYL1/TNNI2/MYL2/TNNC1/ATP2A1/TCAP | 10 |
| BP | GO:0090092 | regulation of transmembrane receptor protein serine/threonine kinase signaling pathway | INHBA/GREM1/PMEPA1/FST/SULF1/TGFBR3/CHRDL1/CILP/GPC3 | 9 |
| BP | GO:0085029 | extracellular matrix assembly | LAMB3/PXDN/COL1A2/MFAP4 | 4 |
| BP | GO:1903900 | regulation of viral life cycle | RSAD2/ISG15/BST2/OASL/IFI27/CXCL8/LAMP3 | 7 |
| BP | GO:2000146 | negative regulation of cell motility | IL24/CXCL13/SERPINE1/BST2/GREM1/THY1/SULF1/PTN/APOD/SLURP1/ADIPOQ | 11 |
| BP | GO:0001676 | long-chain fatty acid metabolic process | PLP1/ALOX12/HPGD/CYP4F12/EPHX2/CYP2C18 | 6 |
| BP | GO:1903169 | regulation of calcium ion transmembrane transport | CXCL10/CXCL9/CXCL11/THY1/ATP1A2/SLN/ATP2A1 | 7 |
| BP | GO:0002544 | chronic inflammatory response | CXCL13/IDO1/CCL11 | 3 |
| BP | GO:0015669 | gas transport | CA2/MB/HBB | 3 |
| BP | GO:0031099 | regeneration | SPP1/POSTN/THY1/NEFL/TGFBR3/PTN/APOD/ENO3 | 8 |
| BP | GO:0034341 | response to interferon-gamma | GBP5/BST2/OASL/CCL20/CYP27B1/CCL18/CCL11/GBP6 | 8 |
| BP | GO:0006959 | humoral immune response | CXCL13/CXCL10/CXCL9/CXCL11/S100A7/CXCL8/CXCL1/CXCL6/SPINK5/CFD/PLA2G2A | 11 |
| BP | GO:0043200 | response to amino acid | COL5A2/COL4A1/COL1A1/COL1A2/RRAGD/PTN | 6 |
| BP | GO:0030500 | regulation of bone mineralization | ISG15/GREM1/CYP27B1/PTN/STATH | 5 |
| BP | GO:0061045 | negative regulation of wound healing | PLAU/SERPINE1/FAP/TSPAN8/ALOX12 | 5 |
| BP | GO:0019373 | epoxygenase P450 pathway | CYP4F12/EPHX2/CYP2C18 | 3 |
| BP | GO:0051797 | regulation of hair follicle development | CDH3/FST/SPINK5 | 3 |
| BP | GO:0030509 | BMP signaling pathway | GREM1/FST/SULF1/TGFBR3/CHRDL1/TMEM100/GPC3 | 7 |
| BP | GO:0010927 | cellular component assembly involved in morphogenesis | ACTA1/MYBPC1/MYL2/ACTN2/TCAP/NEB | 6 |
| BP | GO:0006067 | ethanol metabolic process | ADH1B/ALDH1A1/ADH7 | 3 |
| BP | GO:0035455 | response to interferon-alpha | IFIT3/BST2/LAMP3 | 3 |
| BP | GO:0051900 | regulation of mitochondrial depolarization | IFI6/ALOX12/MYOC | 3 |
| BP | GO:0034612 | response to tumor necrosis factor | AIM2/POSTN/CXCL8/COL1A1/CCL20/CCL18/CCL11/GSDME/TNFRSF12A/ADIPOQ | 10 |
| BP | GO:0090257 | regulation of muscle system process | DSG2/ATP1A2/MYH7/SLN/TNNI2/MYL2/TNNC2/TNNC1/ATP2A1 | 9 |
| BP | GO:0010466 | negative regulation of peptidase activity | MMP9/SERPINE1/IFI6/BST2/LAMP3/SPINK5/SPINK7/A2ML1/GPC3 | 9 |
| BP | GO:0002063 | chondrocyte development | PTHLH/COL11A1/SULF1/VIT | 4 |
| BP | GO:0051279 | regulation of release of sequestered calcium ion into cytosol | CXCL10/CXCL9/CXCL11/THY1/ATP1A2 | 5 |
| BP | GO:0019079 | viral genome replication | RSAD2/ISG15/BST2/OASL/IFI27/CXCL8 | 6 |
| BP | GO:0003254 | regulation of membrane depolarization | IFI6/GPD1L/ALOX12/MYOC | 4 |
| BP | GO:0051882 | mitochondrial depolarization | IFI6/ALOX12/MYOC | 3 |
| BP | GO:0010469 | regulation of signaling receptor activity | PLAU/SERPINE1/GREM1/NEFL/SLURP1/ADH7/ACTN2 | 7 |
| BP | GO:0071772 | response to BMP | GREM1/FST/SULF1/TGFBR3/CHRDL1/TMEM100/GPC3 | 7 |
| BP | GO:0071773 | cellular response to BMP stimulus | GREM1/FST/SULF1/TGFBR3/CHRDL1/TMEM100/GPC3 | 7 |
| BP | GO:0045682 | regulation of epidermis development | CDH3/FST/CYP27B1/CTSL/SPINK5 | 5 |
| BP | GO:0048545 | response to steroid hormone | SPP1/PMEPA1/NEFL/CA2/COL1A1/DSG2/ATP1A2/ACTA1/PTN/ALDH3A1/ADIPOQ | 11 |
| BP | GO:0019369 | arachidonic acid metabolic process | ALOX12/CYP4F12/EPHX2/CYP2C18 | 4 |
| BP | GO:0031103 | axon regeneration | SPP1/NEFL/PTN/APOD | 4 |
| BP | GO:0090101 | negative regulation of transmembrane receptor protein serine/threonine kinase signaling pathway | GREM1/PMEPA1/FST/TGFBR3/CHRDL1/CILP | 6 |
| BP | GO:0001942 | hair follicle development | CDH3/INHBA/FST/TGM3/SPINK5 | 5 |
| BP | GO:0046697 | decidualization | SPP1/CYP27B1/PTN | 3 |
| BP | GO:0060343 | trabecula formation | GREM1/COL1A1/TGFBR3 | 3 |
| BP | GO:0043903 | regulation of interspecies interactions between organisms | RSAD2/ISG15/BST2/OASL/IFI27/CXCL8/LAMP3/CXCL6 | 8 |
| BP | GO:0042742 | defense response to bacterium | CXCL13/SERPINE1/ISG15/S100A7/CCL20/CXCL6/SPINK5/GBP6/PLA2G2A/STATH | 10 |
| BP | GO:0032970 | regulation of actin filament-based process | BST2/PDPN/CCL11/DSG2/ATP1A2/SCIN/MYOC/TNNC1/ATP2A1/ACTN2/NEB | 11 |
| BP | GO:0003206 | cardiac chamber morphogenesis | SEMA3C/COL11A1/TGFBR3/MYH7/MYL2/TNNC1 | 6 |
| BP | GO:0022404 | molting cycle process | CDH3/INHBA/FST/TGM3/SPINK5 | 5 |
| BP | GO:0022405 | hair cycle process | CDH3/INHBA/FST/TGM3/SPINK5 | 5 |
| BP | GO:0098773 | skin epidermis development | CDH3/INHBA/FST/TGM3/SPINK5 | 5 |
| BP | GO:0032461 | positive regulation of protein oligomerization | MMP1/MMP3/AIM2 | 3 |
| BP | GO:0042537 | benzene-containing compound metabolic process | TDO2/IDO1/CYP4B1 | 3 |
| BP | GO:0006979 | response to oxidative stress | MMP3/MMP9/S100A7/NCF2/COL1A1/PXDN/APOD/MB/CA3/ADIPOQ/GPX3/HBB | 12 |
| BP | GO:0003231 | cardiac ventricle development | SEMA3C/COL11A1/TGFBR3/MYH7/MYL2/TNNC1 | 6 |
| BP | GO:0055013 | cardiac muscle cell development | MYBPC1/MYL2/ACTN2/TCAP/NEB | 5 |
| BP | GO:0010524 | positive regulation of calcium ion transport into cytosol | CXCL10/CXCL9/CXCL11/THY1 | 4 |
| BP | GO:0070206 | protein trimerization | COL1A1/COL1A2/ADIPOQ/SCARA5 | 4 |
| BP | GO:1904645 | response to amyloid-beta | MMP3/MMP13/MMP12/MMP9 | 4 |
| BP | GO:0046849 | bone remodeling | SPP1/CTHRC1/GREM1/CA2/PTN | 5 |
| BP | GO:0042476 | odontogenesis | SERPINE1/INHBA/CA2/COL1A1/COL1A2/PAX9 | 6 |
| BP | GO:0055007 | cardiac muscle cell differentiation | GREM1/MYBPC1/MYL2/ACTN2/TCAP/NEB | 6 |
| BP | GO:0045992 | negative regulation of embryonic development | COL5A2/COL5A1/SULF1 | 3 |
| BP | GO:0030510 | regulation of BMP signaling pathway | GREM1/FST/SULF1/CHRDL1/GPC3 | 5 |
| BP | GO:0051899 | membrane depolarization | IFI6/ATP1A2/GPD1L/ALOX12/MYOC | 5 |
| BP | GO:0072593 | reactive oxygen species metabolic process | MMP3/IFI6/NCF2/PXDN/FOXM1/ALOX12/GPX3/EPHX2/HBB | 9 |
| BP | GO:0008217 | regulation of blood pressure | POSTN/COL1A2/ATP1A2/ADIPOQ/CYP4F12/EPHX2/HBB | 7 |
| BP | GO:0002026 | regulation of the force of heart contraction | ATP1A2/MYH7/MYL2 | 3 |
| BP | GO:1901623 | regulation of lymphocyte chemotaxis | CXCL13/CXCL10/S100A7 | 3 |
| BP | GO:0070167 | regulation of biomineral tissue development | ISG15/GREM1/CYP27B1/PTN/STATH | 5 |
| BP | GO:0110149 | regulation of biomineralization | ISG15/GREM1/CYP27B1/PTN/STATH | 5 |
| BP | GO:0042743 | hydrogen peroxide metabolic process | MMP3/PXDN/GPX3/HBB | 4 |
| BP | GO:0042634 | regulation of hair cycle | CDH3/FST/SPINK5 | 3 |
| BP | GO:0042730 | fibrinolysis | PLAU/SERPINE1/FAP | 3 |
| BP | GO:0017001 | antibiotic catabolic process | PXDN/GPX3/ADH7/HBB | 4 |
| BP | GO:0051146 | striated muscle cell differentiation | CXCL10/CXCL9/GREM1/ACTA1/MYBPC1/MYL2/ACTN2/TCAP/NEB | 9 |
| BP | GO:0045071 | negative regulation of viral genome replication | RSAD2/ISG15/BST2/OASL | 4 |
| BP | GO:0006942 | regulation of striated muscle contraction | DSG2/ATP1A2/MYH7/MYL2/ATP2A1 | 5 |
| BP | GO:0018149 | peptide cross-linking | FN1/TGM3/SPRR3/TGM1 | 4 |
| BP | GO:1904064 | positive regulation of cation transmembrane transport | CXCL10/CXCL9/CXCL11/THY1/ATP2A1/ACTN2 | 6 |
| BP | GO:0010769 | regulation of cell morphogenesis involved in differentiation | SPP1/POSTN/FN1/SEMA3C/THY1/PDPN/NEFL/TNFRSF12A/MYOC | 9 |
| BP | GO:0034440 | lipid oxidation | PLA2G7/APOD/ADIPOQ/ALOX12/ADH7 | 5 |
| BP | GO:0010522 | regulation of calcium ion transport into cytosol | CXCL10/CXCL9/CXCL11/THY1/ATP1A2 | 5 |
| BP | GO:0044319 | wound healing, spreading of cells | MMP12/COL5A1/PDPN | 3 |
| BP | GO:0090075 | relaxation of muscle | ATP1A2/SLN/ATP2A1 | 3 |
| BP | GO:0090505 | epiboly involved in wound healing | MMP12/COL5A1/PDPN | 3 |
| BP | GO:0010951 | negative regulation of endopeptidase activity | MMP9/SERPINE1/IFI6/BST2/LAMP3/SPINK5/SPINK7/A2ML1 | 8 |
| BP | GO:0031032 | actomyosin structure organization | ACTA1/MYBPC1/MYL2/MYOC/ACTN2/TCAP/NEB | 7 |
| BP | GO:0042744 | hydrogen peroxide catabolic process | PXDN/GPX3/HBB | 3 |
| BP | GO:0070570 | regulation of neuron projection regeneration | SPP1/THY1/PTN | 3 |
| BP | GO:0090504 | epiboly | MMP12/COL5A1/PDPN | 3 |
| BP | GO:0060389 | pathway-restricted SMAD protein phosphorylation | INHBA/GREM1/PMEPA1/TGFBR3 | 4 |
| BP | GO:0006569 | tryptophan catabolic process | TDO2/IDO1 | 2 |
| BP | GO:0042436 | indole-containing compound catabolic process | TDO2/IDO1 | 2 |
| BP | GO:0046218 | indolalkylamine catabolic process | TDO2/IDO1 | 2 |
| BP | GO:0048021 | regulation of melanin biosynthetic process | CDH3/TYRP1 | 2 |
| BP | GO:0051901 | positive regulation of mitochondrial depolarization | ALOX12/MYOC | 2 |
| BP | GO:0060346 | bone trabecula formation | GREM1/COL1A1 | 2 |
| BP | GO:0010837 | regulation of keratinocyte proliferation | CDH3/SLURP1/TGM1 | 3 |
| BP | GO:0050673 | epithelial cell proliferation | MMP12/CDH3/FAP/SULF1/CCL11/KRT4/TGFBR3/PTN/SLURP1/TGM1/GPC3 | 11 |
| BP | GO:0050729 | positive regulation of inflammatory response | SERPINE1/GBP5/PLA2G7/IDO1/CCL18/PLA2G2A | 6 |
| BP | GO:0031667 | response to nutrient levels | CXCL10/SPP1/MMP7/POSTN/SLC16A1/COL1A1/CYP27B1/RRAGD/PTN/ALDH3A1/ADIPOQ/DAPL1 | 12 |
| BP | GO:0050727 | regulation of inflammatory response | MMP3/MMP9/SERPINE1/GBP5/PLA2G7/IDO1/CCL18/APOD/ADIPOQ/PLA2G2A | 10 |
| BP | GO:0030858 | positive regulation of epithelial cell differentiation | SERPINE1/CYP27B1/ADIPOQ/TMEM100 | 4 |
| MF | GO:0005201 | extracellular matrix structural constituent | LAMC2/COL5A2/POSTN/CTHRC1/LAMB3/COL5A1/FN1/TGFBI/COL4A1/COL10A1/COL1A1/COL11A1/PXDN/COL1A2/COL4A6/DPT/CILP/ADIPOQ/MFAP4/STATH | 20 |
| MF | GO:0005125 | cytokine activity | IL24/CXCL13/CXCL10/INHBA/SPP1/CXCL9/CXCL11/GREM1/CXCL8/CXCL1/CCL20/CCL18/CCL11/CXCL6/FAM3B/FAM3D/SLURP1/ADIPOQ | 18 |
| MF | GO:0008009 | chemokine activity | CXCL13/CXCL10/CXCL9/CXCL11/CXCL8/CXCL1/CCL20/CCL18/CCL11/CXCL6 | 10 |
| MF | GO:0042379 | chemokine receptor binding | CXCL13/CXCL10/CXCL9/CXCL11/CXCL8/CXCL1/CCL20/CCL18/CCL11/CXCL6 | 10 |
| MF | GO:0005539 | glycosaminoglycan binding | LAMC2/CXCL13/CXCL10/MMP7/CXCL11/POSTN/COL5A1/FN1/NELL2/COL11A1/SULF1/CXCL6/TGFBR3/PTN/PCOLCE2/VIT | 16 |
| MF | GO:0008201 | heparin binding | LAMC2/CXCL13/CXCL10/MMP7/CXCL11/POSTN/COL5A1/FN1/NELL2/COL11A1/CXCL6/TGFBR3/PTN/PCOLCE2 | 14 |
| MF | GO:0048018 | receptor ligand activity | IL24/PTHLH/CXCL13/CXCL10/INHBA/SPP1/CXCL9/CXCL11/SEMA3C/GREM1/CXCL8/CXCL1/CCL20/CCL18/CCL11/CXCL6/FAM3B/FAM3D/PTN/SLURP1/ADIPOQ/ENDOU | 22 |
| MF | GO:0030546 | signaling receptor activator activity | IL24/PTHLH/CXCL13/CXCL10/INHBA/SPP1/CXCL9/CXCL11/SEMA3C/GREM1/CXCL8/CXCL1/CCL20/CCL18/CCL11/CXCL6/FAM3B/FAM3D/PTN/SLURP1/ADIPOQ/ENDOU | 22 |
| MF | GO:0030020 | extracellular matrix structural constituent conferring tensile strength | COL5A2/COL5A1/COL4A1/COL10A1/COL1A1/COL11A1/COL1A2/COL4A6 | 8 |
| MF | GO:0008307 | structural constituent of muscle | MYBPC1/MYL1/MYL2/MYOT/ACTN2/TCAP/MYLPF/NEB | 8 |
| MF | GO:0045236 | CXCR chemokine receptor binding | CXCL13/CXCL10/CXCL9/CXCL11/CXCL8 | 5 |
| MF | GO:1901681 | sulfur compound binding | LAMC2/CXCL13/CXCL10/MMP7/CXCL11/POSTN/COL5A1/FN1/NELL2/FST/COL11A1/CXCL6/TGFBR3/PTN/PCOLCE2 | 15 |
| MF | GO:0005178 | integrin binding | ISG15/SPP1/FAP/COL5A1/FN1/TGFBI/THY1/PTPRZ1/PTN/TSPAN8/ACTN2 | 11 |
| MF | GO:0004222 | metalloendopeptidase activity | MMP1/MMP10/MMP3/MMP13/MMP12/MMP9/MMP7/FAP/MMP11 | 9 |
| MF | GO:0020037 | heme binding | TDO2/IDO1/CYP27B1/PXDN/MB/CYP3A5/CYP4B1/CYP4F12/HBB/CYP2C18 | 10 |
| MF | GO:0019825 | oxygen binding | TDO2/MB/CYP3A5/CYP4B1/HBB/CYP2C18 | 6 |
| MF | GO:0005126 | cytokine receptor binding | CXCL13/CXCL10/INHBA/CXCL9/CXCL11/GREM1/CXCL8/CXCL1/CCL20/PXDN/CCL18/CCL11/CXCL6/TGFBR3 | 14 |
| MF | GO:0046906 | tetrapyrrole binding | TDO2/IDO1/CYP27B1/PXDN/MB/CYP3A5/CYP4B1/CYP4F12/HBB/CYP2C18 | 10 |
| MF | GO:0048407 | platelet-derived growth factor binding | COL5A1/COL4A1/COL1A1/COL1A2 | 4 |
| MF | GO:0005518 | collagen binding | MMP13/MMP12/MMP9/FN1/TGFBI/CTSL/PCOLCE2 | 7 |
| MF | GO:0070330 | aromatase activity | CYP3A5/CYP4B1/CYP4F12/CYP2C18 | 4 |
| MF | GO:0050839 | cell adhesion molecule binding | CDH3/ISG15/SPP1/FAP/POSTN/COL5A1/FN1/TGFBI/THY1/MYO1B/TENM2/PTPRZ1/DSG2/PTN/TSPAN8/PPL/ACTN2 | 17 |
| MF | GO:0008237 | metallopeptidase activity | MMP1/MMP10/MMP3/MMP13/MMP12/MMP9/MMP7/FAP/MMP11/CLCA4 | 10 |
| MF | GO:0008236 | serine-type peptidase activity | MMP1/MMP3/MMP9/PLAU/MMP7/FAP/TMPRSS11B/CFD/PRSS27/ENDOU | 10 |
| MF | GO:0016825 | hydrolase activity, acting on acid phosphorus-nitrogen bonds | MMP1/MMP3/MMP9/PLAU/MMP7/FAP/TMPRSS11B/CFD/PRSS27/ENDOU | 10 |
| MF | GO:0017171 | serine hydrolase activity | MMP1/MMP3/MMP9/PLAU/MMP7/FAP/TMPRSS11B/CFD/PRSS27/ENDOU | 10 |
| MF | GO:0043394 | proteoglycan binding | COL5A1/FN1/FST/CTSL/PTN | 5 |
| MF | GO:0001664 | G protein-coupled receptor binding | CXCL13/CXCL10/CXCL9/CXCL11/CTHRC1/CXCL8/CXCL1/CCL20/CCL18/CCL11/CXCL6/MYOC | 12 |
| MF | GO:0004252 | serine-type endopeptidase activity | MMP1/MMP3/MMP9/PLAU/MMP7/FAP/TMPRSS11B/CFD/PRSS27 | 9 |
| MF | GO:0004497 | monooxygenase activity | CYP27B1/FMO2/CYP3A5/CYP4B1/CYP4F12/TYRP1/CYP2C18 | 7 |
| MF | GO:0019838 | growth factor binding | CXCL13/COL5A1/COL4A1/COL1A1/PXDN/COL1A2/TGFBR3/PTN | 8 |
| MF | GO:0004175 | endopeptidase activity | MMP1/MMP10/MMP3/MMP13/MMP12/MMP9/PLAU/MMP7/FAP/MMP11/CTSL/TMPRSS11B/CFD/PRSS27 | 14 |
| MF | GO:0051015 | actin filament binding | MYO1B/MYH2/MYBPC1/MYH7/TNNC2/SCIN/TNNC1/ACTN2/NEB | 9 |
| MF | GO:0016712 | oxidoreductase activity, acting on paired donors, with incorporation or reduction of molecular oxygen, reduced flavin or flavoprotein as one donor, and incorporation of one atom of oxygen | CYP3A5/CYP4B1/CYP4F12/CYP2C18 | 4 |
| MF | GO:0031432 | titin binding | MYBPC1/ACTN2/TCAP | 3 |
| MF | GO:0061134 | peptidase regulator activity | SERPINE1/BST2/FN1/MAL/SPINK5/SPINK7/PCOLCE2/A2ML1/GPC3 | 9 |
| MF | GO:0048020 | CCR chemokine receptor binding | CXCL13/CCL20/CCL18/CCL11 | 4 |
| MF | GO:0005506 | iron ion binding | CYP27B1/CYP3A5/CYP4B1/ALOX12/CYP4F12/TF/CYP2C18 | 7 |
| MF | GO:0016705 | oxidoreductase activity, acting on paired donors, with incorporation or reduction of molecular oxygen | CYP27B1/FMO2/CYP3A5/CYP4B1/CYP4F12/TYRP1/CYP2C18 | 7 |
| MF | GO:0000146 | microfilament motor activity | MYO1B/MYH2/MYH7 | 3 |
| MF | GO:0003779 | actin binding | MYO1B/MYH2/MYBPC1/MYH7/TNNI2/MYL2/TNNC2/MYOT/SCIN/TNNC1/ACTN2/NEB | 12 |
| MF | GO:0016702 | oxidoreductase activity, acting on single donors with incorporation of molecular oxygen, incorporation of two atoms of oxygen | TDO2/IDO1/ALOX12 | 3 |
| MF | GO:0016701 | oxidoreductase activity, acting on single donors with incorporation of molecular oxygen | TDO2/IDO1/ALOX12 | 3 |
| MF | GO:0016803 | ether hydrolase activity | ALOX12/EPHX2 | 2 |
| MF | GO:0018455 | alcohol dehydrogenase [NAD(P)+] activity | ADH1B/ADH7 | 2 |
| MF | GO:0019911 | structural constituent of myelin sheath | MAL/PLP1 | 2 |
| MF | GO:0048019 | receptor antagonist activity | FST/PXDN/ADH7 | 3 |
| MF | GO:0015643 | toxic substance binding | CYP4B1/EPHX2 | 2 |
| MF | GO:0016801 | hydrolase activity, acting on ether bonds | ALOX12/EPHX2 | 2 |
| MF | GO:0036122 | BMP binding | GREM1/TCAP | 2 |
| MF | GO:1902936 | phosphatidylinositol bisphosphate binding | MYO1B/PLEK2/GSDME/SCIN/ACTN2 | 5 |
| MF | GO:0017022 | myosin binding | ACTA1/MYL2/MYOC/AMPD1 | 4 |
| MF | GO:0030898 | actin-dependent ATPase activity | MYO1B/MYH7 | 2 |
| MF | GO:0032036 | myosin heavy chain binding | MYL2/AMPD1 | 2 |
| MF | GO:0016504 | peptidase activator activity | FN1/MAL/PCOLCE2 | 3 |
| MF | GO:0016709 | oxidoreductase activity, acting on paired donors, with incorporation or reduction of molecular oxygen, NAD(P)H as one donor, and incorporation of one atom of oxygen | CYP27B1/FMO2/CYP4F12 | 3 |
| MF | GO:0005546 | phosphatidylinositol-4,5-bisphosphate binding | MYO1B/GSDME/SCIN/ACTN2 | 4 |
| CC | GO:0062023 | collagen-containing extracellular matrix | MMP9/LAMC2/SERPINE1/COL5A2/POSTN/CTHRC1/S100A7/LAMB3/COL5A1/FN1/TGFBI/GREM1/COL4A1/COL10A1/COL1A1/COL11A1/PXDN/SULF1/PTPRZ1/COL1A2/CTSL/COL4A6/DPT/CILP/PTN/ADIPOQ/MYOC/MFAP4/VIT/GPC3 | 30 |
| CC | GO:0098644 | complex of collagen trimers | COL5A2/COL5A1/COL4A1/COL1A1/COL11A1/COL1A2/COL4A6 | 7 |
| CC | GO:0043292 | contractile fiber | IDO1/MYH2/ACTA1/MYBPC1/MYH7/MYL1/TNNI2/MYL2/TNNC2/MYOT/TNNC1/ACTN2/TCAP/MYLPF/NEB | 15 |
| CC | GO:0005581 | collagen trimer | COL5A2/CTHRC1/COL5A1/COL4A1/COL10A1/COL1A1/COL11A1/COL1A2/COL4A6/ADIPOQ | 10 |
| CC | GO:0005583 | fibrillar collagen trimer | COL5A2/COL5A1/COL1A1/COL11A1/COL1A2 | 5 |
| CC | GO:0098643 | banded collagen fibril | COL5A2/COL5A1/COL1A1/COL11A1/COL1A2 | 5 |
| CC | GO:0030017 | sarcomere | MYH2/ACTA1/MYBPC1/MYH7/MYL1/TNNI2/MYL2/TNNC2/MYOT/TNNC1/ACTN2/TCAP/NEB | 13 |
| CC | GO:0030016 | myofibril | MYH2/ACTA1/MYBPC1/MYH7/MYL1/TNNI2/MYL2/TNNC2/MYOT/TNNC1/ACTN2/TCAP/NEB | 13 |
| CC | GO:0016459 | myosin complex | MYO1B/MYH2/MYH7/CGNL1/MYL1/MYL2/MYLPF | 7 |
| CC | GO:0005604 | basement membrane | LAMC2/LAMB3/COL5A1/FN1/TGFBI/COL4A1/COL4A6/PTN | 8 |
| CC | GO:0005788 | endoplasmic reticulum lumen | SPP1/COL5A2/COL5A1/FN1/COL4A1/COL10A1/COL1A1/COL11A1/COL1A2/COL4A6/CHRDL1/TF/GPC3 | 13 |
| CC | GO:0005859 | muscle myosin complex | MYH2/MYH7/MYL1/MYLPF | 4 |
| CC | GO:0005865 | striated muscle thin filament | ACTA1/MYBPC1/TNNI2/TNNC2/TNNC1 | 5 |
| CC | GO:0036379 | myofilament | ACTA1/MYBPC1/TNNI2/TNNC2/TNNC1 | 5 |
| CC | GO:0045177 | apical part of cell | FAP/BST2/FN1/THY1/PDPN/MYO1B/CA2/DSG2/MAL/CLCA4/RHCG/SCNN1B/CYP4F12/TF | 14 |
| CC | GO:0016460 | myosin II complex | MYH2/MYH7/MYL1/MYLPF | 4 |
| CC | GO:0001533 | cornified envelope | DSC1/DSG2/PPL/SPRR3/SCEL/TGM1 | 6 |
| CC | GO:0016324 | apical plasma membrane | BST2/FN1/THY1/PDPN/DSG2/MAL/CLCA4/RHCG/SCNN1B/CYP4F12/TF | 11 |
| CC | GO:0031258 | lamellipodium membrane | FAP/PDPN/PLEK2 | 3 |
| CC | GO:0032982 | myosin filament | MYH2/MYBPC1/MYH7 | 3 |
| CC | GO:0030057 | desmosome | DSC1/DSG2/PPL | 3 |
| CC | GO:0030018 | Z disc | MYBPC1/MYH7/MYOT/ACTN2/TCAP/NEB | 6 |
| CC | GO:1904724 | tertiary granule lumen | MMP9/CXCL1/CRISP3/HBB | 4 |
| CC | GO:0031674 | I band | MYBPC1/MYH7/MYOT/ACTN2/TCAP/NEB | 6 |
| CC | GO:0030175 | filopodium | PDPN/MYO1B/TENM2/ACTA1/ACTN2 | 5 |
| CC | GO:0043256 | laminin complex | LAMC2/LAMB3 | 2 |
| CC | GO:0031093 | platelet alpha granule lumen | SERPINE1/FN1/CFD/ACTN2 | 4 |
| CC | GO:0070820 | tertiary granule | MMP9/PLAU/DSC1/CXCL1/CRISP3/HBB | 6 |
